# N-Terminal Region of Gelsolin Induces Apoptosis of Activated Hepatic Stellate Cells by a Caspase-Dependent Mechanism

**DOI:** 10.1371/journal.pone.0044461

**Published:** 2012-08-29

**Authors:** Budhaditya Mazumdar, Keith Meyer, Ranjit Ray

**Affiliations:** 1 Department of Internal Medicine, Saint Louis University, St. Louis, Missouri, United States of America; 2 Department of Molecular Microbiology and Immunology, Saint Louis University, St. Louis, Missouri, United States of America; Children's Hospital Boston & Harvard Medical School, United States of America

## Abstract

Activated hepatic stellate cells (HSCs) are the major source for alteration of extracellular matrix in fibrosis and cirrhosis. Conditioned medium (CM) collected from immortalized human hepatocytes (IHH) have earlier been shown to be responsible for apoptosis of HSCs. In this study, we have shown that antibodies raised against a peptide derived from a linear B-cell epitope in the N-terminal region of gelsolin identified a gelsolin fragment in IHH CM. Analysis of activated stellate cell death by CM collected from Huh7 cells transfected with plasmids encoding gelsolin deletion mutants suggested that the N-terminal half of gelsolin contained sequences which were responsible for stellate cell death. Further analysis determined that this activity was restricted to a region encompassing amino acids 1–70 in the gelsolin sequence; antibody directed to an epitope within this region was able to neutralize stellate cell death. Gelsolin modulation of cell death using this fragment involved upregulation of TRAIL-R1 and TRAIL-R2, and involved caspase 3 activation by extrinsic pathway. The apoptotic activity of N-terminal gelsolin fragments was restricted to activated but not quiescent stellate cells indicating its potential application in therapeutic use as an anti-fibrotic agent. Gelsolin fragments encompassing N-terminal regions in polypeptides of different molecular sizes were detected by N-terminal peptide specific antiserum in IHH CM immunoprecipitated with chronically HCV infected patient sera, suggesting the presence of autoantibodies generated against N-terminal gelsolin fragments in patients with chronic liver disease.

## Introduction

HSCs are located in perisinusoidal space which contains several extracellular matrix (ECM) molecules, such as type I, III, IV, V and VI collagens, laminin, fibronectin and proteoglycans [1], [2]. ECM molecules are recognized as the primary cellular source of matrix components in chronic liver disease, and therefore play a critical role in the development and maintenance of liver fibrosis [3]. In quiescent state, HSCs have a low mitotic activity and are mainly responsible for the uptake, storage and delivery of retinoids [4]. However, in response to liver injury conditions, these cells undergo an activation process to transform into proliferative, fibrogenic, proinflammatory, and contractile myofibroblasts which express α-smooth muscle actin (α-SMA) [4]. These activated stellate cells secrete extracellular matrix protein type I collagen that is associated with the development of liver fibrosis and cirrhosis [5], [6]. Hepatic fibrosis is reversible [7]–[9], and its resolution requires the loss of activated HSCs via apoptosis [7], [8], [10].

We studied the functional relationship between IHH generated by the introduction of HCV core gene into primary human hepatocytes, and human hepatic stellate cells (LX2) spontaneously generated as immortalized phenotype in cell culture. Apoptosis of activated LX2 cells occurred in the presence of CM from IHH [11]. Further studies suggested that IHH CM increased the expression of TRAIL receptors on LX2 cell surface and induced apoptosis by a caspase dependent mechanism. Peptide mass fingerprinting of a purified soluble mediator from CM indicated that gelsolin fragments may play a role in LX2 apoptosis [11]. Gelsolin, an ∼83 KD calcium-binding protein, is involved in the remodeling of cellular actin filaments associated with cell shape changes and movement. Gelsolin interacts with actin in a Ca^2+^-dependent manner and weakens noncovalent bonds between actin filaments to make them susceptible to cleavage [12], [13]. Gelsolin sequence contains six structurally homologous domains (S1 through S6) of 120–130 amino acids that appear to have originated from gene triplication of the prototypical domain followed by gene duplication [14], [15]. Hence, gelsolin is composed of two domains (the N-terminal S1–S3 and the C-terminal S4–S6 halves) separated by a 70 amino acid linker sequence, which is cleaved by different proteases [16]–[18].

Besides playing an important role in remodeling of actin filaments inside cells, gelsolin is also secreted from several mammalian cell types into blood. Originally defined by its interactions with actin, this secretory form, now called plasma gelsolin, circulates in mammalian blood at concentrations of 200–300 µg/ml [19]–[22]. Human plasma gelsolin differs from the cytoplasmic isoform by an additional sequence of 24 amino acids, designated as the “plasma extension” signal that remains in the mature protein after cleavage of the peptide that directs plasma gelsolin to the secretory pathway into the endoplasmic reticulum, where plasma gelsolin folds and a disulfide bond is formed [23], [24]. Liver has been suggested to be a major source of plasma gelsolin and human hepatoma cell line HepG2 produces and secretes plasma gelsolin [23]. Plasma gelsolin acts as an actin-scavenging protein to prevent increases in blood viscosity caused by considerable amounts of actin that are released from dying cells during inflammatory processes, especially during acute lung injury [25]. A single amino acid mutation in domain 2 (S2) of plasma gelsolin (D187N/Y) affects Ca^2+^ binding and folding of plasma gelsolin [26]. This leads to aberrant cleavage of the misfolded protein in the trans-Golgi, generating a secretory 68 kD fragment of the protein (C68) [27], [28]. C68 can be further cleaved into smaller fragments by extracellular proteases like MT1-MMP [29] to generate major (8 kD) and minor (5 kD) amyloidogenic fragments that form extracellular membranous deposits on various muscle as well as non-muscle tissues [30], [31].

Over-expression of the Ca^2+^ independent severing N-terminal half of gelsolin induces apoptosis, whereas gelsolin null neutrophils have a delayed onset of apoptosis [16]. In this study, we have identified the N-terminal region comprising of amino acids 1–70 in the gelsolin sequence as responsible for apoptotic cell death of activated HSCs involving an upregulation of TRAIL-R1 and TRAIL-R2 and activation of caspase 3 by extrinsic pathway.

## Materials and Methods

### Cells

IHH were generated by transfection of a plasmid DNA expressing HCV core genomic region of genotype 1a (Genbank accession number M62321) into primary human hepatocytes under the control of a CMV promoter [32], [33]. LX2 cells [11], [34] was used in this study and grown in **Dulbecco's modified Eagle medium (DMEM)**, supplemented with 10% fetal bovine serum and 2X L-glutamine. Human hepatoma (Huh7) cells were grown in DMEM, supplemented with 10% fetal bovine serum and antibiotics. Cell proliferation was detected using a CellTiter 96 Aqueous non-radioactive cell proliferation assay (Promega, Madison., WI), following supplier's protocol. LX2 cells were grown on Matrigel (BD Biosciences) when necessary to maintain the quiescent phenotype.

### Gelsolin

A cDNA encoding human gelsolin was procured (Origene). Purified gelsolin from human plasma was purchased (Sigma, St. Louis).

### Cloning and expression of N-terminal gelsolin fragments

Human plasma gelsolin cDNA (encompassing amino acids 1–782) was cloned into a N-terminal FLAG-bearing pCMV vector (D1) that was subcloned by restriction digestion to yield plasmid DNA constructs encoding N-terminal (1–450) (D2) and C-terminal (450–782) (D3) fragments. Plasmids encoding smaller N-terminal fragments (1–70; D2–F1) (1–130; D2–F2) and (70–450; D2–F3) were further subcloned from the plasmid encoding larger FLAG-tagged (1–450; D2) fragment using digestion by specific restriction enzymes followed by ligation. Plasmid DNAs were introduced into Huh 7 cells by transfection using lipofectamine for protein expression.

### Preparation of conditioned medium (CM)

IHH were grown on a collagen type I coated on plastic plate in small airway epithelial cell growth medium (SAGM) supplemented with 5% chemically denatured fetal bovine serum at 37°C. At ∼90% confluency, cells were washed and incubated in serum free SAGM for 48 h. CM was clarified by centrifugation at 6,000 g to remove cell debris, supplemented with 2X L-glutamine, and stored at −20°C until use.

### Antibody generation

Chicken antisera were generated from predicted N-terminal linear B-cell epitopes and evaluated for characterization of reactivity with gelsolin. Antisera were purified and used in subsequent experiments for detection of N-terminal gelsolin fragment. The antisera G1 and G2 raised against two peptides derived from N-terminal region of gelsolin (representing amino acids 35–50: CSQAGAPQGRVPEARPN, and 75–93: CKFDLVPVPTNLYGDFFTGD, respectively) differed in their reactivity against IHH CM by Western blot analysis.

### Protein expression

Western blot analysis was performed to analyze the activation levels of caspases-9 and 3 using specific monoclonal antibodies (R&D Systems, Minneapolis, MN). CM from IHH or CHO cells transfected with plasmids encoding specific gelsolin fragments were incubated with anti-TRAIL-R1, anti-TRAIL-R2 (R & D Systems) or isotype specific antibodies (negative controls), followed by FITC-conjugated secondary antibody for fluorescence-activated–cell-sorter (FACS) analysis. Non-specific background was determined from untreated and isotype matched unrelated negative control antibodies. Positive cells were identified by FACScan (Beckton Dickinson), and results analyzed using Cell Quest software. Ten thousand cells were analyzed for each sample and gating was set on the basis of a dot plot for 90° light scatter versus forward angle light scatter to exclude dead cells and debris from analysis.

## Results

### Identification of N-terminal gelsolin fragments in conditioned medium from hepatocyte cultures

**Figure 1:**
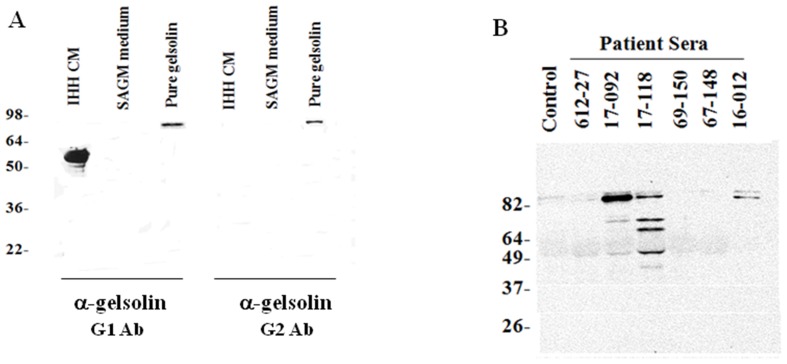
Presence of N-terminal gelsolin fragments in hepatocyte conditioned medium. **Panel A:** Western blot of CM with chicken antibodies raised against peptides (G1 and G2) selected from the N-terminal region of gelsolin. Purified gelsolin from human plasma was included as a positive control in the experiment. SAGM medium, used for culture of stellate cells, was used as a negative control. (B) Immunoprecipitation of CM with healthy control and chronically HCV infected patient sera, followed by Western blotting with chicken anti-gelsolin antibody G1. The positions of protein molecular weight markers (kD) are shown on the left.

Previous reports indicated that immunoprecipitation of IHH CM, responsible for apoptotic activity towards HSCs, with sera from chronic liver patients followed by Western blotting with a gelsolin specific monoclonal antibody GS-2C4 (Sigma) directed to the C-terminal half of the protein, revealed several cleaved fragments presumably originating from gelsolin (ranging from 30 kD to 65 kD) [11]. It is likely that the N-terminal half of the protein, reported to possess apoptotic activity [16] will escape detection by this antibody. In order to detect the N-terminal half in gelsolin fragments present in CM, chicken antibodies G1 and G2 were generated that were directed to peptides designed from two adjacent regions (amino acid residues 35–50 and 75–93, respectively) in the N-terminal half of gelsolin. Western blot analysis suggested that these two antibodies differed in their reactivity to IHH CM resulting in different band patterns (Fig. 1, panel A). Full-length gelsolin was detected by both the antibodies. However, only G1 antibody could detect the ∼60 kD gelsolin fragment in IHH CM. Gelsolin fragments were also identified in CM by Western blot using gelsolin G1 antibody after immunoprecipitation with sera from patients with chronic hepatitis (Fig. 1, panel B). Gelsolin antibody G1 was subsequently used for detection of gelsolin fragments in this study.

### Stellate cell death associates with N-terminal region of gelsolin

**Figure 2:**
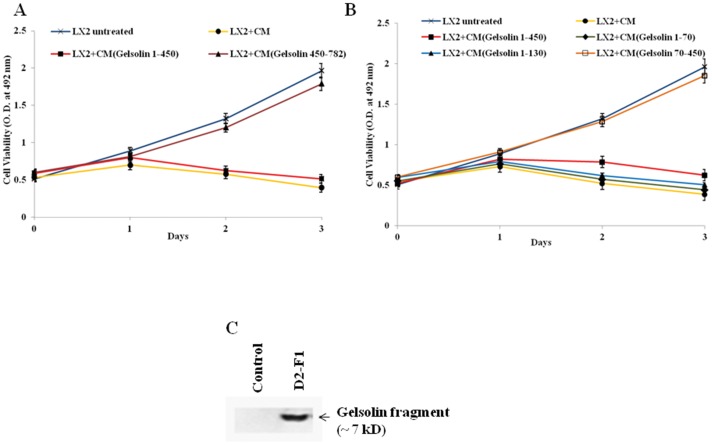
Effect of CM from Huh 7 cells transfected with gelsolin fragments on LX2 growth. **Panel A:** CM from Huh7 cells transfected with plasmid constructs- D2 encoding N-terminal half (amino acids 1–450) and D3 encoding C-terminal half (amino acids 450–782) displayed distinct LX2 cell viability. LX2 cells treated with IHH CM as a positive control and untreated LX2 as a negative control were included in the experiment. Cell viability was determined from triplicate culture wells by a CellTiter 96 Aqueous non-radioactive cell proliferation assay (Promega) at different time points and presented as mean and standard deviations. **Panel B:** LX2 cell viability following incubation with CM from Huh7 cells transfected with deletion mutants encoding gelsolin fragments (amino acids 1–450, 1–70, 1–130 and 70–450) were similarly determined. Effect of medium (untreated) and IHH CM treated positive controls were included for comparison. Cell viability was determined from triplicate culture wells by a CellTiter 96 Aqueous non-radioactive cell proliferation assay (Promega) at different time points and presented as mean and standard deviations. **Panel C**: Expression of gelsolin N-terminal 1–70 fragment in CM from 293T cells transfected with FLAG-tagged D2–F1 construct. CM from transfected cells was immunoprecipitated with anti-FLAG antibody and analyzed by Western blot using anti-gelsolin G1 antibody. CM from mock-transfected 293T cells served as negative control.

Conditioned medium from Huh7 cells transfected with plasmid construct D2 encoding N-terminal half of gelsolin sequence (amino acids 1–450) induced rapid human hepatic stellate cell death as determined using a Cell proliferation assay kit (Promega). However, stellate cell death was not detectable when incubated with CM from Huh7 cells transfected with plasmid construct D3 encoding gelsolin C-terminal half (amino acids 450–782) (Fig. 2, panel A). Conditioned medium from non-transfected Huh7 cells acted as a negative control, and did not cause a detectable stellate cell death. Next, we compared the ability of differently-sized N-terminal gelsolin fragments to cause stellate cell death. Conditioned medium collected from Huh7 cells after introduction of either one of the two smaller N-terminal gelsolin fragments- D2–F1 (1–70) or D2–F2 (1–130) showed marginally stronger stellate cell death activity compared to the larger D2 fragment (1–450) fragment from which they are derived with the 1–70 being the most potent among the three of them (Fig. 2, panel B). On the other hand, CM collected from Huh7 cells after introduction of gelsolin fragment D2–F3 (70–450) did not cause an appreciable difference in stellate cell death as compared to untreated control indicating that stellate cell death activity of IHH CM could be restricted to 1–70 region in gelsolin sequence. For the purpose of detecting smaller gelsolin fragments in CM from cells transfected with corresponding gene constructs, 293T cells were transfected with the N-terminal FLAG-tagged D2–F1 construct that encodes gelsolin sequence 1–70. CM was collected, immunoprecipitated with anti-FLAG antibody, and subsequently detected using anti-gelsolin G1 antibody. The gelsolin fragment was detected as a ∼7 kD band (Fig. 2, panel C). 293T cells were chosen over Huh7 cells for this purpose because of consistently higher levels of expression and detection of gelsolin fragments in these cells after transfection of corresponding gene constructs.

### N-terminal gelsolin fragment mediated stellate cell death is antagonized by peptide specific antibody

**Figure 3:**
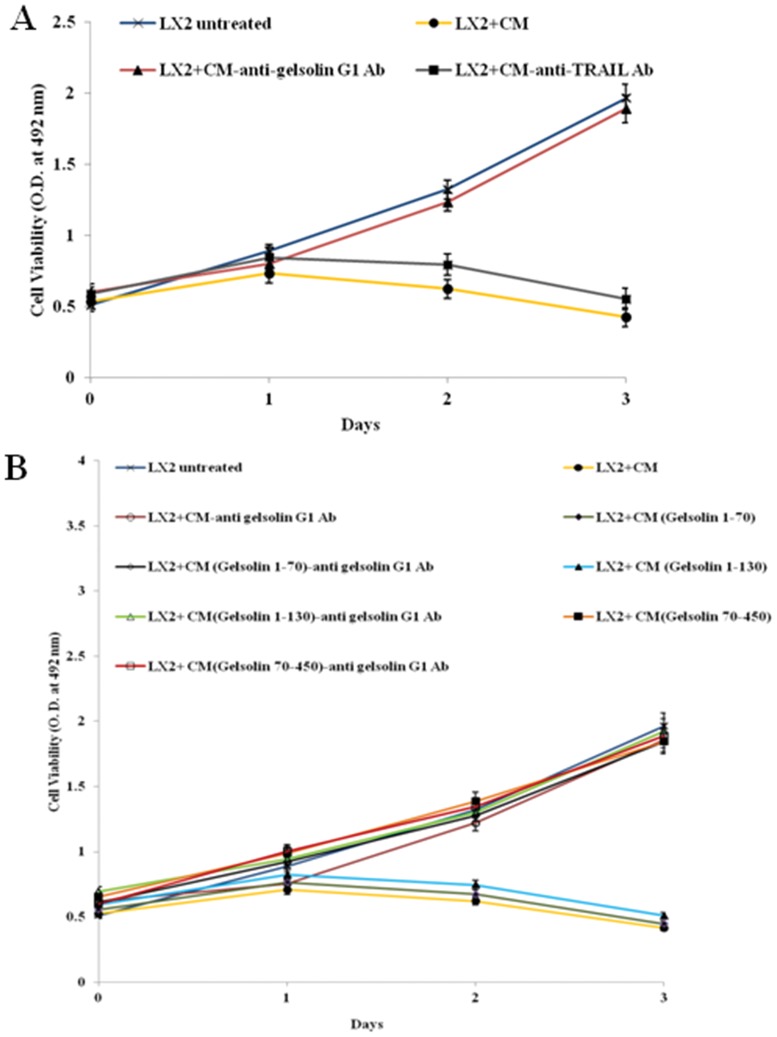
Antibody to N-terminal fragment of gelsolin antagonizes LX2 cell death. CM from IHH (**panel A**) or from Huh 7 cells transfected with N-terminal gelsolin fragments consisting of amino acids 1–70, 1–130 and 70–450 (**panel B**) was preincubated with immobilized gelsolin G1 or TRAIL antibody, and subsequently tested for LX2 cell growth. Effect of normal medium (untreated) and CM without preincubation were included for comparison. Cell viability was determined from triplicate culture wells by a CellTiter 96 Aqueous non-radioactive cell proliferation assay (Promega) at different time points and presented as mean and standard deviations.

We have shown previously that CM utilizes the TRAIL signaling pathway to induce apoptosis [11]. Initial microscopic observations suggested that onset of stellate cell death by IHH CM is delayed for about 24 h after incubation with immobilized anti-gelsolin G1 antibody. This was further examined by quantifying LX2 cell death after adding CM with or without neutralization with the anti-gelsolin G1 antibody as well as anti-TRAIL antibody over a 72 h time period. For this, the antibodies were immobilized on Protein G Sepharose beads, and CM was incubated with the immobilized antibody. The suspension was centrifuged to separate the beads, filter sterilized, and added to LX2 cells for apoptotic cell death. LX2 cell viability was significantly affected after 24 h in presence of IHH CM. On the other hand, viability was restored to almost similar levels as that in untreated controls when CM was preincubated with G1 antibody (Fig. 3, panel A). On the other hand, neutralization with anti-TRAIL antibody did not cause any significant inhibition of the ability of IHH CM in stellate cell death. Next, we determined G1 antibody specific antagonism of LX2 cell death after incubation of CM from Huh 7 cells transfected with multiple N-terminal gelsolin fragments consisting of amino acids 1–70, 1–130 and 70–450 respectively. Inhibition of stellate cell death was observed by the G1 antibody against fragments of gelsolin encompassing the amino acid residues 1–70, and 1–130; as well as the fragment derived from gelsolin containing supernatant (Fig. 3, panel B). Thus, the experiments with N-terminal specific antibodies validated the role of gelsolin in IHH CM-mediated LX2 stellate cell death.

### N-terminal gelsolin fragment induces TRAIL-R1 and TRAIL-R2 expression on stellate cell surface

**Figure 4:**
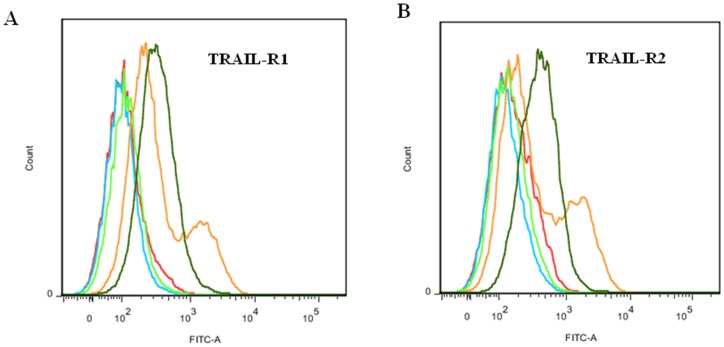
Induction of TRAIL receptors by N-terminal region in gelsolin fragments. FACS analysis was performed to determine the expression levels of TRAIL-R1 (**panel A**) and TRAIL-R2 (**panel B**) on LX2 cell surface treated with CM from Huh 7 cells transfected with gelsolin fragments 1–70 (dark green line), 1–450 (orange line) and 450–782 (red line). Expression of these proteins in cells treated with media (blue line) was included as control. Cells were either treated with specific monoclonal antibody or isotype control antibody (light green line), followed by treatment with FITC-conjugated secondary antibody for FACS analysis.

Previous studies using FACS analyses indicated that IHH CM mediated stellate cell death involves significant upregulation of TRAIL-R1 and TRAIL-R2 expression on cell surface [11]. In this study, we used FACS analysis for TRAIL-R1 and TRAIL-R2 expression on stellate cells exposed to CM containing gelsolin deletion mutants to determine whether this upregulation was mediated by the N-terminal region of gelsolin fragments present in CM. We observed that upregulation of both TRAIL-R1 (Fig. 4, panel A) and TRAIL-R2 (Fig. 4, panel B) by CM from Huh 7 cells transfected with constructs encoding the N-terminal half of gelsolin sequence (1–450) as well as smaller N-terminal (1–70) fragment. The C-terminal half of gelsolin (450–782) did not cause an induction of TRAIL-R expression.

### N-terminal gelsolin fragment induces caspase-3 mediated apoptotic death in activated LX2 cells

**Figure 5:**
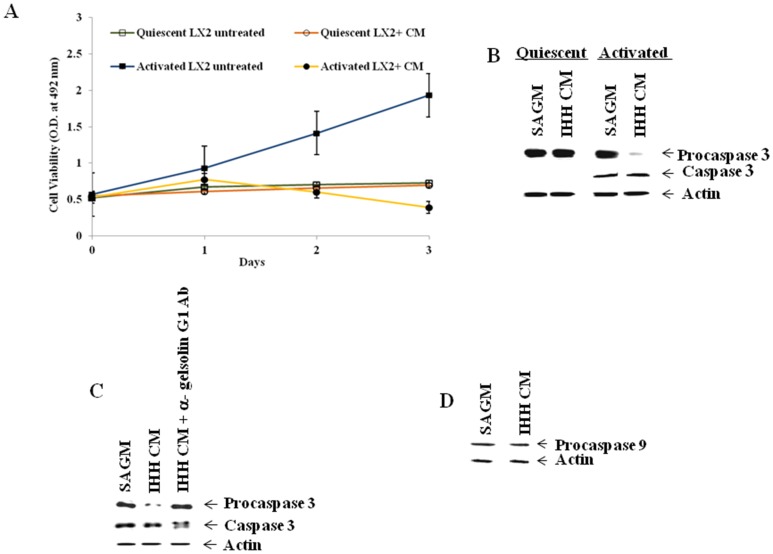
Activated but not quiescent LX2 cells undergo caspase 3 mediated apoptosis in presence of IHH CM. **Panel A**: Comparative study of cell viability between quiescent and activated LX2 cells in the presence of IHH CM. Effect of normal medium (untreated) used for both quiescent and activated LX2 cells was included for comparison. Cell viability was determined from triplicate culture wells by a CellTiter 96 Aqueous non-radioactive cell proliferation assay (Promega) at different time points and presented as mean and standard deviations. **Panel B**: Expression of pro- and cleaved caspase 3 in quiescent and activated LX2 cells incubated with IHH CM. **Panel C**: LX2 cells incubated with CM with or without prior treatment with immobilized anti-gelsolin G1 antibody. **Panel D**: Expression of procaspase 9 in LX2 cells in the presence of IHH CM. Unused medium (SAGM) was included as a negative control in these experiments. Cellular actin was used as an internal control to verify the level of protein load in each lane.

Activated LX2 cells, when grown on Matrigel-coated plate, tend to revert to the quiescent phenotype. In order to achieve quiescent state, stellate cells were grown on Matrigel and microscopically monitored for a change in appearance. The quiescent state of the stellate cells was also examined by staining for desmin and smooth muscle actin (SMA) by Western blotting. These protein markers were absent or present at a very low level in quiescent state, and their levels dramatically enhanced upon activation following growth on plastic surface. Quiescent LX2 cells on Matrigel were incubated with IHH CM and cell viability was assayed by the cell proliferation assay kit (Promega). Unlike activated LX2 cells, quiescent LX2 cell death was not observed in the presence of IHH CM, even after 3 days (Fig 5, panel A). This observation was further supported by unchanged procaspase 3 levels in quiescent LX2 cells that were incubated with IHH CM compared to those in media control (Fig. 5, panel B). On the other hand, a significant reduction in procaspase-3 expression in activated LX2 cells was observed when incubated with IHH CM. Thus, our results suggested that activated - but not quiescent- stellate cells undergo apoptosis in the presence of IHH CM. Interestingly, caspase 3 activation was decreased when activated stellate cells were incubated with IHH CM previously treated with immobilized anti-gelsolin G1 antibody (Fig. 5, panel C). The results validate that apoptotic activity of IHH CM upon stellate cells is directed by the N-terminal region in gelsolin present in the CM. Apoptosis of activated stellate cells in IHH CM did not involve procaspase 9 activation (Fig. 5, panel D), indicating that the intrinsic pathway is not involved in the process.

## Discussion

Quiescent stellate cells undergo activation to adopt myofibroblast morphology and secrete type I collagen, the principal matrix protein responsible for the development of liver fibrosis and cirrhosis under pathological conditions. Our previous report has shown that conditioned medium from IHH caused apoptosis of activated stellate cells, and gelsolin was implicated as an active component in IHH CM [11]. In this study, we have shown that the apoptotic activity of gelsolin resides in the N-terminal region present in the S1 domain. We have investigated the possible mechanism of action for apoptotic activity of gelsolin fragment and have further shown the involvement of TRAIL-R1 and R2 upregulation and activation of caspases 3 by the extrinsic pathway.

Fragments derived from gelsolin sequence have been identified previously in IHH CM by immunoprecipitation followed by Western blot analysis using commercially available gelsolin monoclonal antibody GS-2C4, directed to its C-terminal half [11]. However, this antibody recognizes an epitope containing a C-terminal actin binding site, and failed to deplete gelsolin from CM or inhibit CM mediated LX2 apoptosis. To understand the gelsolin fragments responsible for CM mediated apoptotic activity towards LX2 cells, we developed an anti-gelsolin antibody that recognize gelsolin fragments in Western blotting and immunoprecipitation, and also antagonizes CM-mediated LX2 apoptosis. N-terminal region of gelsolin comprising of first three homologous domains (S1–S3) has been previously shown to be apoptotic [16]. We generated chicken antibody G1 against a synthetic peptide derived from the region comprising amino acids 35–50 in the N-terminal half of gelsolin. Also, we performed subcloning of a construct encoding full-length gelsolin (amino acids 1–782) or smaller deletion mutants cloned into a pCMV mammalian expression vector. Quantitative estimation of stellate cell death mediated by CM from Huh7 cells transfected with the gelsolin fragments indicated higher apoptotic potential of the N-terminal half of gelsolin compared to the C-terminal half. Also, comparison of stellate cell killing activity among the N-terminal fragments revealed that the apoptotic activity displayed by gelsolin fragments was localized in the N-terminal region encoding amino acids 1–70.

Next, we investigated whether anti-peptide G1 antibody representing a B cell epitope of the N-terminal region of gelsolin antagonizes stellate cell death mediated by CM containing N-terminal gelsolin fragments 1–450 and 1–70 from transfected Huh7 cells. We observed a significant inhibition of stellate cell death by these N-terminal gelsolin fragments when CM was preincubated with this antibody indicating the functional significance of N-terminal gelsolin fragments. Previous studies [11] indicated that IHH CM mediated LX2 cell death involved upregulation of TRAIL-R1 and TRAIL-R2 on the cell surface. We observed a similar upregulation of TRAIL-R1 and TRAIL-R2 expression levels on LX2 cell surface upon exposure to CM from Huh7 cells containing N-terminal gelsolin fragments representing amino acid residues 1–450 or 1–70. Interestingly, TRAIL did not appear to act as a soluble mediator in the process as evident by the absence of TRAIL in CM by Western blotting as well as failure of anti-TRAIL antibody to effectively inhibit CM-mediated LX2 cell death. LX2 apoptosis by CM containing gelsolin fragments involved activation of procaspase 3 and inhibited to normal level when CM was incubated with anti-gelsolin G1 antibody, further indicating that N-terminal region of gelsolin fragments in CM leads to apoptosis of LX2 by caspase activation. Unaltered levels of procaspase 9 suggested that the apoptotic pathway involved the extrinsic route involving TRAIL receptor upregulation. The mechanism regulating gelsolin-mediated upregulation of TRAIL receptors in a TRAIL-independent manner is intriguing and is still largely unknown. Sequence homology or structural similarity between gelsolin and TRAIL were not observed to warrant a direct interaction between gelsolin and TRAIL receptors. There are also no reports for specific gelsolin receptors on cell membrane surfaces. Gelsolin is susceptible to cleavage to amyloidogenic fragments of sizes as small as 6–8 kDs that deposit on membranous surfaces of many tissues [29]–[31], and will be interesting to determine whether this phenomenon activates apoptotic pathway on the target cell.

Interestingly, apoptotic cell death by IHH CM, was directed to activated LX2 cells, but did not affect quiescent LX2 cells. Administration of gelsolin can be potentially employed as an agent to resolve fibrosis, characterized by death of activated stellate cells in a suitable animal model. In this study, we have provided evidence for induction of cell death by a generated fragment of gelsolin which mimics the activity apparent in stellate cells treated with CM from immortalized hepatic cell lines. Further studies should elucidate precise mechanism of cell death, and identify therapeutic potential of this gelsolin fragment as an anti-fibrotic agent.
